# Breast density in dedicated breast computed tomography: Proposal of a classification system and interreader reliability

**DOI:** 10.1097/MD.0000000000025844

**Published:** 2021-05-07

**Authors:** Jann Wieler, Nicole Berger, Thomas Frauenfelder, Magda Marcon, Andreas Boss

**Affiliations:** Institute of Diagnostic and Interventional Radiology, University Hospital Zurich, University of Zurich, Zurich, Switzerland.

**Keywords:** breast density, interrater reliability, spiral breast-CT

## Abstract

The aim of this study was to develop a new breast density classification system for dedicated breast computed tomography (BCT) based on lesion detectability analogous to the ACR BI-RADS breast density scale for mammography, and to evaluate its interrater reliability.

In this retrospective study, 1454 BCT examinations without contrast media were screened for suitability. Excluding datasets without additional ultrasound and exams without any detected lesions resulted in 114 BCT examinations. Based on lesion detectability, an atlas-based BCT density (BCTD) classification system of breast parenchyma was defined using 4 categories. Interrater reliability was examined in 40 BCT datasets between 3 experienced radiologists.

Among the included lesions were 63 cysts (55%), 18 fibroadenomas (16%), 7 lesions of fatty necrosis (6%), and 6 breast cancers (5%) with a median diameter of 11 mm. X-ray absorption was identical between lesions and breast tissue; therefore, the lack of fatty septae was identified as the most important criteria for the presence of lesions in glandular tissue. Applying a lesion diameter of 10 mm as desired cut-off for the recommendation of an additional ultrasound, an atlas of 4 BCTD categories was defined resulting in a distribution of 17.5% for density A, 39.5% (B), 31.6% (C), and 11.4% (D) with an intraclass correlation coefficient (ICC) among 3 readers of 0.85 to 0.87.

We propose a dedicated atlas-based BCTD classification system, which is calibrated to lesion detectability. The new classification system exhibits a high interrater reliability and may be used for the decision whether additional ultrasound is recommended.

**Key Points**

- Presentation of image examples for the estimation of breast density in dedicated breast computed tomography.

## 1 Introduction

Breast cancer is the most common cancer diagnosed in women with an incidence of 12.3% in the normal population with a median age at diagnosis of 61 years and a increasing trend (incidence of 9.09% in the 1970s).^[1]^ Mammography screening is known to reduce mortality in breast cancer, and it has been estimated that the combination of a mammography screening program with adjuvant therapy results in a relative reduction in mortality of 37%.^[2]^ Apart from genetic disposition, hormonal influences such as estrogen replacement therapy and oral contraception are discussed as potential risk factors. Moreover, mammographic density (MD) was demonstrated as an important risk factor for the development of breast cancer.^[3]^ Women with extremely dense breast tissue demonstrate a 2- to 6-fold increase of breast cancer risk.^[4]^

MD is defined as the relative amount of glandular tissue based on the mammographic appearance of fibroglandular tissue, which is not inferable from physical examination.^[5]^ In MD also changes in breast density due to different hormonal levels as menopausal transition and aging are reflected. A standardized reporting system for MD was introduced according to the ACR BI-RADS (American College of Radiology Breast Imaging-Reporting and Data System) catalogue last updated in 2013, classifying MD in 4 categories from “A” to “D,” with category A denoting almost completely fatty tissue, B denoting scattered fibroglandular tissue, C indicating heterogeneously dense tissue and D representing extremely dense tissue.^[6]^ Overall, 40% of women have MD of ACR BI-RADS C or D, in women below 50 years dense MD is found in more than half of the patients.^[7]^

Apart from its meaning for the estimation of breast cancer risk, MD is important for the assessment of the diagnostic performance of mammography screening. For low MD, a sensitivity of mammography for the detection of breast cancer of 87% to 98% is reported, which substantially drops to 30% to 63% in extremely dense breasts.^[8–11]^ In patients with mammographic dense breasts, supplemental ultrasound may increase the detection rate for breast cancer,^[8,12]^ which is reflected in many guidelines recommending additional ultrasound in ACR BI-RADS densities “C” and “D.”^[13]^

Recently, cone-beam and spiral breast-CT using photon-counting detector technology has been introduced as a new truly 3D breast imaging technology.^[14]^ The advantages of breast-CT (BCT) over mammography are the increased patient comfort (no breast compression needed),^[15]^ the possibility of multiplanar reconstructions with a high isotropic spatial resolution, and the lack of super-imposing glandular tissue^[16]^ at comparable radiation dose to screening mammographies in spiral BCT.^[17]^ Cone-beam breast-CT is reported to have a significant higher dose than mammography.^[18,19]^ The sensitivity of BCT in dense MD was reported to be higher compared to 2D mammography.^[19,20]^ The high isotropic spatial resolution allows 3 dimensional analysis and density measures of detected lesions and contrast-enhanced BCT might be an alternative modality for patients with contraindications to MR mammography.^[21–23]^

The existing studies on BCT regarding lesion detectability and BCTD use the ACR BI-RADS atlas to describe MD in BCT.^[19,20,24]^ This existing atlas has been developed for mammography and does not take into consideration the true 3D nature of the images in BCT. The lack of superposition of breast tissue in BCT might have an influence on the perceived density. In this study, we analyzed BCTD in correlation to lesion detectability, created a dedicated atlas-based 4-level density scale for the use in BCT analogous to the ACR BI-RADS density for mammography, and evaluated the proposed scale regarding interrater reliability. The main motivation was to allow an estimation of the sensitivity of each individual BCT study for future use and to help radiologists take a decision whether additional ultrasound is necessary in asymptomatic patients.

## 2 Materials and method

### 2.1 Patient selection

A retrospective analysis of patient data in our local radiologic information system was performed, which was approved by the local ethics committee. Informed consent was waived for this retrospective study. All the BCT examinations at our institution performed between August 2018 and October 2019 were selected independently of the indication for imaging (727 exams consisting of 2 sides, therefore 1454 breast exams). Only exams with additional ultrasound on the same day were further evaluated (N = 523) and the radiologic report analyzed regarding presence of any described lesion. Exams without lesions or very small lesions (<=6 mm) were excluded (N = 409). A total of 114 BCT examinations were finally evaluated. Mean age of this final patient cohort was 54 years (36–80 years) with median age of 52 years. Additional demographic data is not routinely documented in the radiologic report or in our picture archiving and communication system and was therefore not further analyzed. In the picture archiving and communication system archive, mammographies of the same patients were searched up to the year 2010 and if available MD was compared to BCTD.

### 2.2 BCT examinations

All patient examinations were performed on a dedicated breast CT imager using spiral image acquisition with a CdTe photon-counting detector with a detector pixel size of (0.1 mm)^2^ and a total detector area of 280 × 50 mm^2^ (nu-view, AB-CT GmbH, Erlangen, Germany). The maximum diameter of the Field-of-View is 190 mm, and the scan length can be adjusted to the values 80, 120, and 160 mm depending of the length of the breast. The x-ray tube exhibits a 0.3 mm focal spot size, and 3-mm Al filtration is applied. A fixed X-ray tube voltage of 60 kV is used, whereas the tube current may be adjusted between 5 mA and 125 mA. In all patients, a tube current of 32 mA was applied. No contrast media was used. The examination scans were acquired in a spiral mode with 2 seconds rotation time, 2000 projections per rotation, except for the first rotation. Image reconstruction was done in standard mode with a soft kernel (Shepp-Logan) at (300 μm)^3^ voxel size with 2 × 2 detector binning using a Feldkamp-type filtered back projection (FBP) algorithm. Breast ultrasound was either performed with handheld ultrasound by a radiologic resident (2 different vendors: GE Logic E9 (N = 20 patients) or Philips iU22 (N = 59 patients) with a linear transducer with at least 11 MHz) or with automated breast ultrasound (Invenia Automated Breast Ultrasound System, General Electric Healthcare, Sunnyvale, CA) using a C 15-6XW reverse curve, 5–14 MHz transducer with an aperture length of 15.3 cm, a transducer travel distance of 16.9 cm, and a depth up to 5 cm (N = 35 patients).

### 2.3 Breast density

Based on the lesion detectability, the BCT examinations were divided in 4 categories with different Breast-CT Density (BCTD):

1. almost no glandular tissue with complete visibility of all the lesions,
2. some glandular tissue with soft tissue lesions larger than 10 mm visible,
3. partially dense breast tissue with lesions of 10 mm potentially not visible,
4. extremely dense tissue with severely limited visibility of lesions.

The goal was to be able to detect 10/10 lesions in density A, at least 8/10 in density B and below 5/10 in density C and D to reflect the high sensitivity of mammography for low BCTD and decreased detection rate in high BCTD. From the categorization, the most typical examples were determined as an atlas guide with 2 different views: **AG1**, coronary plane, raw images from the BCT (0.3 mm slice thickness), and **AG2**, axial images, multiplane reformatted images (3 mm slice thickness averaged, mpr).

### 2.4 Interrater reliability

The proposed atlas guide was evaluated regarding interrater reliability by selecting 40 example BCT examinations regardless of the presence of lesions with different levels of BCTD. These exemplary BCT studies were selected by the main author solely based on their perceived density. Three radiologists, all with extensive experience in the assessment of BCT examinations, were asked to categorize these 40 cases regarding BCTD using the proposed atlas guide. Each reader evaluated each exam:

1. using AG1 (raw images, coronal plane), and
2. using AG2 (mpr, axial plane).

The intraclass correlation coefficient (ICC) between the 3 readers using 4 discrete categories and Cohen kappa between every pair of readers using only 2 categories (A/B vs C/D) were calculated using SPSS (IBM SPSS Statistics version 25). According to Kundel and Polansky and Landis and Koch, an ICC greater than 0.80 was considered to be indicative of “almost perfect agreement (ICC = 1, “perfect agreement”).^[25,26]^ To assess differences in the median of the classification results of the cohort among the 3 readers, the rank-based non-parametric Kruskal–Wallis H test for comparison of 3 groups has been applied to both readings AG1 and AG2 as well as to the decision whether additional ultrasound is required (density A/B vs C/D in AG1 and AG2) with a significance level of 0.05.

## 3 Results

### 3.1 Lesion characteristics

One hundred fourteen lesions were included: 63 cysts (55%), 18 fibroadenomas (16%), 7 lesions of fatty necrosis (6%), 6 breast cancers (5%), 14 undetermined lesions with benign sonographic features (12%), and 6 various soft tissue lesions (5%). Mean size of the lesions was 13 mm (7–39 mm) with a median size of 11 mm. Overall 40 lesions were below 10 mm, 50 lesions were between 10 to 15 mm and 24 lesions were larger than 15 mm.

### 3.2 Establishment of a BCTD atlas

**Figure 1:**
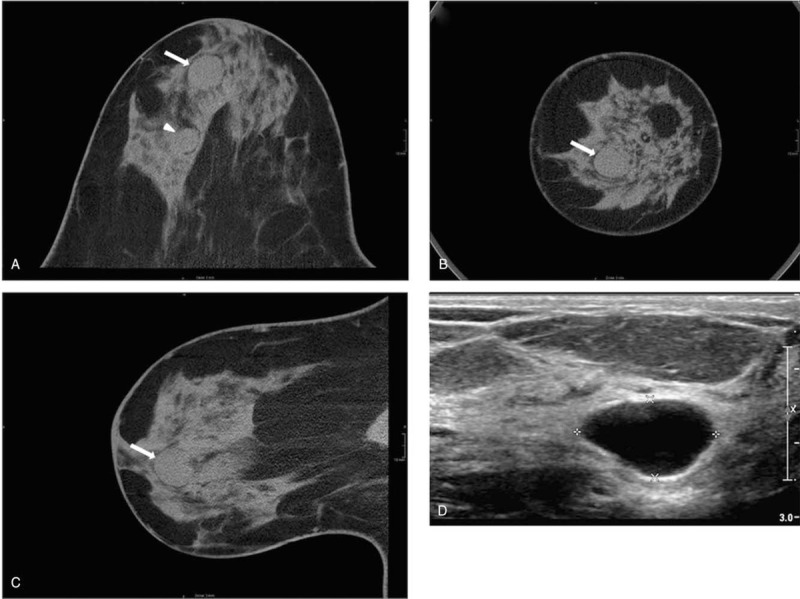
BCT slices axial (A), coronal (B) and sagittal (C) plane and corresponding ultrasound (D). Cystic lesion (arrow, 17 mm) visible even in relatively dense BCTD due to fatty septae. Note the second, smaller lesion in a (arrowhead, 14 mm).

**Figure 2:**
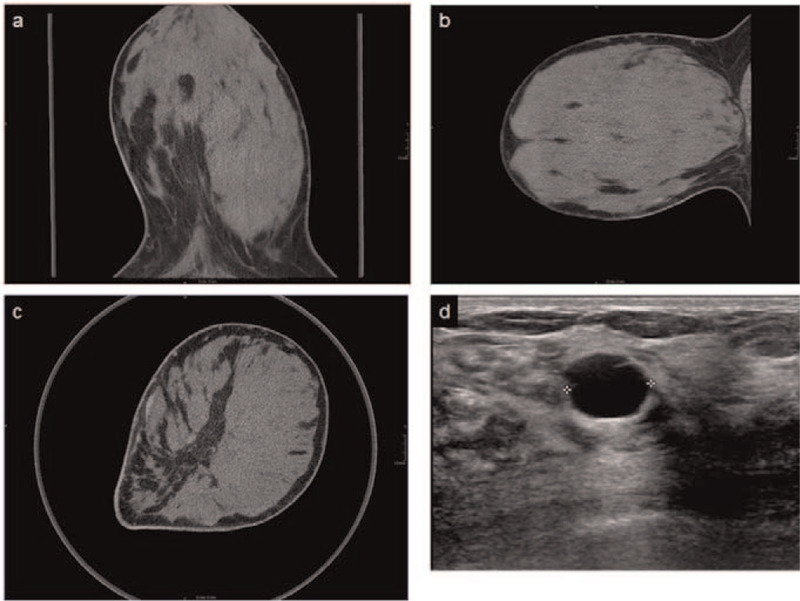
BCT slices axial (A), coronal (B) and sagittal (C) plane and corresponding ultrasound (D). Sonographic proven cyst (12 mm) not visible in BCT in a patient with very high BCTD without fatty septae in the densest parenchymal areas.

**Figure 3:**
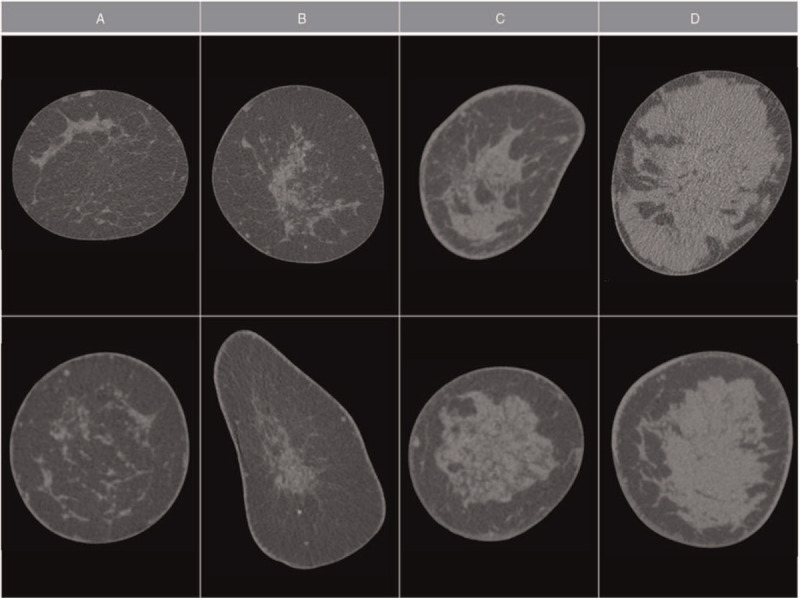
Atlas guide 1 (**AG1**), coronal plane, raw images with 0.3 mm slice thickness. Breast density classification from low density (A) to high density (D) with 2 examples for each category.

**Figure 4:**
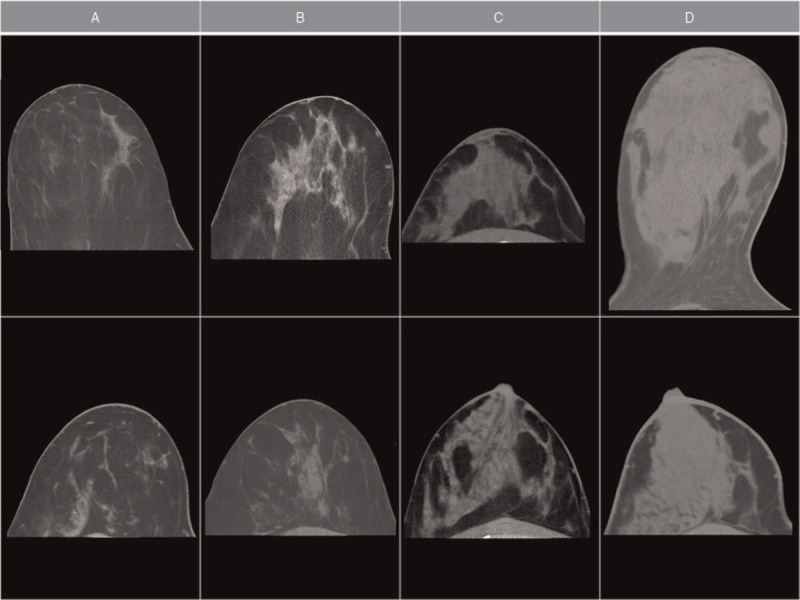
Atlas guide 2 (**AG2**), axial plane, multiplane reformatted images with 3 mm averaged slice thickness. Breast density classification from low density (A) to high density (D) with 2 examples for each category.

Overall 79 of 114 lesions were detectable in BCT (69% of all lesions). Typical examples of lesions are provided in Figures 1 and 2. In low BCTD the lesions were easily detectable whereas in high BCTD lesions were often obscured by surrounding dense breast tissue. There was no relevant density contrast neither between glandular tissue and breast lesions, nor among different types of breast lesions. However even in relatively dense breast tissue the presence of small septae of fatty tissue between the fibroglandular structures enabled an exclusion of lesions larger than 10 mm because both, soft tissue lesions and cystic lesions lack the presence of fatty septae within their structures. This feature was used as the main criterion to distinguish BCTD categories B (presence of fatty septae between fibroglandular tissue even in the densest areas of the breast) and C (absence of fatty septae between fibroglandular tissue in dense spots of glandular tissue larger than 10 mm). Besides lesion visibility the most dense part of the breast tissue was evaluated, as in the ACR BI-RADS catalogue regarding MD. Two atlas guides with image examples of 4 levels of BCTD were created based on the above described features, giving 2 examples for every density level in 2 different planes. Atlas guide AG1 is provided in Figure 3 with coronary raw images of standard BCT images with 0.3 mm slice thickness, and atlas guide AG2 is demonstrated in Figure 4 with multiplane reformatted images of 3 mm slice thickness.

### 3.3 Breast density distribution and lesion detectability

Applying the 2 BCTD guides AG1 and AG2, the following distribution of breast densities was present in the cohort of 114 patient examinations: 20 (17.5%) were estimated as density A, 45 (39.5%) as density B, 36 (31.6%) as density C and 13 (11.4%) as density D. Overall in 31 patients a previous mammography was available. In 13 patients (42%) MD was the same as the BCTD and in 18 patients (52%) MD was 1 class higher as evaluated with BCT. In patients below 50 years (N = 43) there were 1 density A, 13 density B, 20 density C and 9 density D. In patients above 60 years (N = 26) there were 10 density A, 13 density B, 2 density C and 1 density D. Patients between 50 and 60 years (N = 45) showed mostly density B and D (Table 1). With these proposed categories of different BCTD 20/20 lesions (100%) in density A, 38/45 lesions (84%) in density B, 15/36 lesions (42%) in density C and 6/13 lesions (46%) in density D were detectable reflecting the high sensitivity of BCT for lesion detection in low BCTD and the decreased sensitivity for higher BCTD. These results are in accordance to the previously stated sensitivities of BCT.^[18–20]^

**Table 1:** Distribution of breast-CT density (BCTD) of the analyzed cohort regarding patient age.

	A	B	C	D	Total
<50 yr	1	13	20	9	43
50–60 yr	9	19	14	3	45
>60 yr	10	13	2	1	26
Total	20	45	36	13	114

### 3.4 Interrater reliability

Three experienced radiologists (JW 2 years of experience in breast imaging, NB 7 years, and AB 15 years) evaluated the 40 BCT examinations using the proposed atlas guides AG1 and AG2 with results shown in Table 2. Intraclass correlation coefficient was 0.87 (95% CI 0.79, 0.92) for AG1 (raw images, coronal plane) and 0.85 (95% CI 0.77, 0.91) for AG2 (mpr, axial plane), reflecting the near perfect interrater correlation.

**Table 2:** Estimation of breast-CT density (BCTD) of different readers using AG1 and AG2 with 40 examplary breast-CT (BCT) exams.

	Reader 1		Reader 2		Reader 3	
	AG1	AG2	AG1	AG2	AG1	AG2
A	9	9	9	9	18	14
B	12	11	13	13	9	4
C	11	12	13	13	7	10
D	8	8	5	5	6	12

The results for the binary evaluation (A/B vs C/D) are shown in Table 3. The kappa-values between the readers using only 2 categories (A/B vs C/D) are demonstrated in Table 4.

**Table 3:** Binary evaluation of breast-CT density (low vs high BCTD) by the different readers using AG1 and AG2 with 40 exemplary breast-CT (BCT) exams.

	Reader 1		Reader 2		Reader 3	
	AG1	AG2	AG1	AG2	AG1	AG2
Low	21	20	22	22	27	18
High	19	20	18	18	13	22

**Table 4:** Kappa-values between each pair of readers using only 2 categories of breast-CT density (low vs high BCTD) for each AG separately. According Landis and Koch 0.6 to 0.8 substantial and >0.8 almost perfect agreement.

AG1	Reader 1	Reader 2	Reader 3
Reader 1		0.85	0.70
Reader 2			0.74
Reader 3			

AG2	Reader 1	Reader 2	Reader 3
Reader 1		0.80	0.9
Reader 2			0.80
Reader 3			

Comparing the 2 atlas guides the results were similar. Subjective evaluation of the presence of fatty septae was reported to be easier in AG1. Reader 3 evaluated the BCTD systematically higher in AG2 as compared to AG1. The use of AG1 was faster and easier because there was no need for post-processing procedures.

In none of the statistical test, a difference in the median of the groups could be identified with all calculated *P* values above the significance level of .05: AG1 with *P* = .16 (H = 3.65), AG 2 with *P* = .81 (H = 0.407), AG1 (A/B vs C/D) with *P* = .46 (H = 1.53), and AG2 (A/B vs C/D) with *P* = .74 (H = 0.60).

## 4 Discussion

In this study, we propose a breast density classification for breast CT examinations based on lesion detectability with fatty septae between glandular tissue being identified as the most important criterion for visibility of potential lesions. Two different atlas guides were evaluated, AG1 based on the raw images of BCT without further postprocessing, and AG2 with axial reformation and averaging of 10 slices; thereby resembling the familiar ACR BIRADS mammography breast density atlas. We were able to demonstrate that both proposed atlas guides exhibit a high reliability with excellent interrater agreement. We identified AG1 as the most suitable atlas guide from the 2 evaluated schemes, mainly because of the advantage that no further postprocessing is required and classification can be carried out directly from the raw images.

BCT is a new and promising modality in breast imaging. The advantages are the high isotropic resolution and patient comfort since there is no need for breast compression. Thirty five perceny of women experience discomfort and up to 6% frank pain during and following mammography^[27]^ and up to 46% of women who do not attend further mammography do so because of pain in their first examination.^[28,29]^ BCT is the only modality that can detect suspicious microcalcifications and soft tissue breast cancers without patient discomfort. Our scanner is the first clinically approved spiral BCT with a single photon-counting detector. In contrast with cone-beam BCT the radiation dose is lower and similar to mammography.^[19,30]^ Even if the sensitivity of BCT is reported to be higher than sensitivity of mammography in women with high MD, missed breast cancers due to superimposed breast tissue is an issue as in mammography.^[19]^ Therefore, a reporting system for BCTD in BCT as a surrogate for the sensitivity of the examination analogous to the ACR BI-RADS 4-level density scale is needed.

MD density has 2 important aspects:

1. MD is an important risk factor for the development of breast cancer with higher MD leading to higher breast cancer risk,
2. MD influences the sensitivity of mammography for the detection of breast cancer with higher MD resulting in lower sensitivity.

For MD, both aspects are not identically correlated to the classification system. Aspect

1. is mostly linked to the *amount* of glandular tissue, whereas aspect
2. is related to the *amount and distribution* of glandular tissue.

The differences have been exposed at the most recent revision of the ACR BIRADS density classification. Before 2013, the amount of glandular tissue in percent in the mammography has been used for the classification of MD. It has been recognized that this form of classification is too rigid for the decision whether additional ultrasound is recommended or not. In the ACR BI-RADS atlas after 2013, the classification has been recalibrated reflecting the unsuitability of quantitative techniques describing now the MD in a Likert scale with example images. Similarly, a new density classification system is required for spiral BCT due to the higher lesion detectability. The most important aspect here is the consideration of fatty septae between glandular tissue, which is not applicable in mammography due to its nature as a projection technique.

For the establishment of the BCTD classification, the decisions for categories A (even very small lesions can be detected due to the lack of glandular tissue) and D (even large lesions can be missed due to the extremely dense glandular tissue) are relatively straight forward. The main calibration of the classification has to be carried out for the distinction of categories B (glandular tissue present but no ultrasound necessary) and C (glandular tissue present requiring supplemental ultrasound). For this reason, we evaluated all available BCT datasets at our institution for lesion detectability within glandular tissue. During the evaluation of lesion visibility and breast density, we noticed that in some patients even with high BCTD the presence of fatty septae between the fibroglandular tissue enabled us to exclude larger lesions. This feature was used to differentiate between BCTD categories B and C using a threshold of 10 mm as the desired cut-off meaning that in BCTD category B a soft tissue lesion of at least 10 mm should be detectable without additional ultrasound examination. From our findings, we extracted example images and the resulting atlas guide AG1, which we propose, is presented in Figure 3. The distribution of BCTD in the 4 categories in the 114 BCT exams were similar to the reported distribution of MD in mammography.^[7]^

Comparing the BCTD to mammographies in those patients, in which a previous mammography had been carried out, a decrease in the density scale was seen in the large majority of cases. This decrease of density may in part be explained because of interval involution of breast tissue. However, Ma et al showed a trend towards higher density classification in cone-beam breast-CT compared to mammography.^[24]^ As we observed a decrease in the BCTD scale, it may be hypothesized that this finding is potentially due to the increased reader-perceived sensitivity of BCT compared to mammography and the respective lower classification in the density scale. Interestingly density distribution varied with patient age which is known in mammography^[7]^ and can be viewed as a quality criterion for the evaluation of BCTD.

In mammography, MD is rated based on a visual scale according to the ACR BI-RADS catalogue, which gives the radiologists some freedom in the decision whether the density and distribution of glandular tissue in the mammography require additional ultrasound. However, such an objective decision making comes at the short-coming of substantial inter-reader and even intra-reader variability.^[31,32]^ The standardization of such a decision using machine learning seem straight forward, and particularly deep convolutional neural networks have been successfully applied to classify MD according to the BI-RADS catalogue.^[33]^ In breast-CT a machine learning approach for classification of BCTD seems also highly desirable though more complicated to achieve due to the large size of the 3-dimensional BCT datasets.

This retrospective study has several limitations: First, a relatively small number of examinations were used to calculate the interrater reliability. A larger cohort might be necessary to confirm the high values of ICC and Kappa using the proposed atlas guide. Second, no analysis regarding different lesion types was performed. Therefore, it remains unclear whether a better atlas guide could be proposed calibrated to the detection of breast cancer alone. Third, no prospective study was carried out, and it remains unclear how the initial decision on supplemental ultrasound without the establishment of an atlas guide for standardization might have influenced the study cohort. Fourth, no correlation of BCTD to mammographic breast density or quantitative breast density assessment was done, which was out of the scope of this study. Fifthly, our study does not evaluate the sensitivity or specificity of breast cancer detection in BCT regarding different levels of BCTD.

## 5 Conclusion

Lesion detection in X-ray based breast imaging is challenging in women with dense MD, which is reflected in guidelines recommending supplemental ultrasound for high MD. Spiral BCT is an interesting new modality not requiring painful breast compression and potentially exhibiting higher sensitivity compared to mammography. A new dedicated BCTD classification system is needed providing a standardization for the decision whether supplemental ultrasound should be performed in conjunction with BCT and to give the referring physician an estimate of the sensitivity of BCT in women with dense breasts. We analyzed 114 BCT exams over a period of 15 months regarding lesion visibility and created a 4-point atlas-based BCTD classification, analogous to the ACR BI-RADS scale for mammography, for future use in BCT. With this classification 90% of the sonographically known lesions were detectable in BCT in low BCTD (A and B) whereas only 43% of lesions were visible in high BCTD (C and D) in our cohort, thus justifying additional ultrasound in high BCTD. The new BCTD classification is easy to use and exhibits high interrater reliability with an intraclass correlation coefficient of over 0.85. This atlas is thought for human readers allowing a visual estimation of BCTD, future research might be done regarding machine learning algorithms and (semi-)quantitative automated analysis of BCTD in spiral BCT.

## Author contributions

**Conceptualization:** Jann Wieler, Andreas Boss.

**Data curation:** Jann Wieler, Nicole Berger, Andreas Boss.

**Formal analysis:** Andreas Boss, Jann Wieler, Nicole Berger.

**Investigation:** Andreas Boss, Jann Wieler.

**Methodology:** Jann Wieler, Andreas Boss.

**Project administration:** Jann Wieler.

**Resources:** Jann Wieler.

**Writing – original draft:** Andreas Boss, Jann Wieler.

**Writing – review & editing:** Andreas Boss, Jann Wieler, Nicole Berger, Magda Marcon, Thomas Frauenfelder.

## References

[1] Advani P Moreno-Aspitia A. Current strategies for the prevention of breast cancer. Breast Cancer (Dove Med Press) 2014;6:59–71. 24833917 10.2147/BCTT.S39114 PMC4018310

[2] Plevritis SK Munoz D Kurian AW. Association of screening and treatment with breast cancer mortality by molecular subtype in US women, 2000-2012. JAMA 2018;319:154–64. 29318276 10.1001/jama.2017.19130 PMC5833658

[3] Boyd NF Guo H Martin LJ. Mammographic density and the risk and detection of breast cancer. N Engl J Med 2007;356:227–36. 17229950 10.1056/NEJMoa062790

[4] Kamangar F Dores GM Anderson WF. Patterns of cancer incidence, mortality, and prevalence across five continents: defining priorities to reduce cancer disparities in different geographic regions of the world. J Clin Oncol 2006;24:2137–50. 16682732 10.1200/JCO.2005.05.2308

[5] Lam PB Vacek PM Geller BM. The association of increased weight, body mass index, and tissue density with the risk of breast carcinoma in Vermont. Cancer 2000;89:369–75. 10918168 10.1002/1097-0142(20000715)89:2<369::aid-cncr23>3.0.co;2-j

[6] Ekpo EU Egbe NO Egom AE. Mammographic breast density:comparison across women with conclusive and inconclusive mammography reports. J Med Imaging Radiat Sci 2016;47:55–9. 31047165 10.1016/j.jmir.2015.10.008

[7] Stomper PC D'Souza DJ DiNitto PA. Analysis of parenchymal density on mammograms in 1353 women 25-79 years old. AJR Am J Roentgenol 1996;167:1261–5. 8911192 10.2214/ajr.167.5.8911192

[8] Berg WA Blume JD Cormack JB. Combined screening with ultrasound and mammography vs mammography alone in women at elevated risk of breast cancer. JAMA 2008;299:2151–63. 18477782 10.1001/jama.299.18.2151 PMC2718688

[9] Boyd NF. Mammographic density and risk of breast cancer. Am Soc Clin Oncol Educ Book 2013. 10.14694/EdBook_AM.2013.33.e57 23714456

[10] Kolb TM Lichy J Newhouse JH. Comparison of the performance of screening mammography, physical examination, and breast US and evaluation of factors that influence them: an analysis of 27,825 patient evaluations. Radiology 2002;225:165–75. 12355001 10.1148/radiol.2251011667

[11] Hollingsworth AB. Redefining the sensitivity of screening mammography: A review. Am J Surg 2019;218:411–8. 30739738 10.1016/j.amjsurg.2019.01.039 PMC6640096

[12] Berg WA Zhang Z Lehrer D. Detection of breast cancer with addition of annual screening ultrasound or a single screening MRI to mammography in women with elevated breast cancer risk. JAMA 2012;307:1394–404. 22474203 10.1001/jama.2012.388 PMC3891886

[13] Wockel A Festl J Stuber T. Interdisciplinary screening, diagnosis, therapy and follow-up of breast cancer. Guideline of the DGGG and the DKG (S3-Level, AWMF Registry Number 032/045OL, December 2017) - part 1 with recommendations for the screening, diagnosis and therapy of breast cancer. Geburtshilfe Frauenheilkd 2018;78:927–48. 30369626 10.1055/a-0646-4522 PMC6202580

[14] Berger N Marcon M Saltybaeva N. Dedicated breast computed tomography with a photon-counting detector initial results of clinical in vivo imaging. Invest Radiol 2019;54:409–18. 30829942 10.1097/RLI.0000000000000552

[15] Li H Yin L He N. Comparison of comfort between cone beam breast computed tomography and digital mammography. Eur J Radiol 2019;120:108674. 31557718 10.1016/j.ejrad.2019.108674

[16] Berger N Marcon M Frauenfelder T. Dedicated spiral breast computed tomography with a single photon-counting detector: initial results of the first 300 women. Invest Radiol 2020;55:68–72. 31592797 10.1097/RLI.0000000000000609

[17] Shim S Saltybaeva N Berger N. Lesion detectability and radiation dose in spiral breast CT with photon-counting detector technology: a phantom study. Invest Radiol 2020. 10.1097/RLI.0000000000000662 32209815

[18] O’Connell A Conover DL Zhang Y. Cone-beam CT for breast imaging: radiation dose, breast coverage, and image quality. AJR Am J Roentgenol 2010;195:496–509. 20651210 10.2214/AJR.08.1017

[19] Wienbeck S Uhlig J Luftner-Nagel S. The role of cone-beam breast-CT for breast cancer detection relative to breast density. Eur Radiol 2017;27:5185–95. 28677053 10.1007/s00330-017-4911-z

[20] He N Wu YP Kong Y. The utility of breast cone-beam computed tomography, ultrasound, and digital mammography for detecting malignant breast tumors: a prospective study with 212 patients. Eur J Radiol 2016;85:392–403. 26781145 10.1016/j.ejrad.2015.11.029

[21] Uhlig J Fischer U Surov A. Contrast-enhanced cone-beam breast-CT: analysis of optimal acquisition time for discrimination of breast lesion malignancy. Eur J Radiol 2018;99:09–16. 10.1016/j.ejrad.2017.12.003 29362157

[22] Wienbeck S Fischer U Perske C. Cone-beam breast computed tomography: CT density does not reflect proliferation potential and receptor expression of breast carcinoma. Transl Oncol 2017;10:599–603. 28666188 10.1016/j.tranon.2017.05.004 PMC5491450

[23] Wienbeck S Fischer U Luftner-Nagel S. Contrast-enhanced cone-beam breast-CT (CBBCT): clinical performance compared to mammography and MRI. Eur Radiol 2018;28:3731–41. 29594402 10.1007/s00330-018-5376-4

[24] Ma Y Cao Y Liu A. A reliability comparison of cone-beam breast computed tomography and mammography: breast density assessment referring to the fifth edition of the BI-RADS atlas. Acad Radiol 2019;26:752–9. 30220584 10.1016/j.acra.2018.07.023

[25] Kundel HL Polansky M. Measurement of observer agreement. Radiology 2003;228:303–8. 12819342 10.1148/radiol.2282011860

[26] Landis JR Koch GG. The measurement of observer agreement for categorical data. Biometrics 1977;33:159–74. 843571

[27] Rutter DR Calnan M Vaile MS. Discomfort and pain during mammography: description, prediction, and prevention. BMJ 1992;305:443–5. 1392955 10.1136/bmj.305.6851.443 PMC1882539

[28] Elwood M McNoe B Smith T. Once is enough--why some women do not continue to participate in a breast cancer screening programme. N Z Med J 1998;111:180–3. 9640316

[29] Whelehan P Evans A Wells M. The effect of mammography pain on repeat participation in breast cancer screening: a systematic review. Breast 2013;22:389–94. 23541681 10.1016/j.breast.2013.03.003

[30] Kalender WA Kolditz D Steiding C. Technical feasibility proof for high-resolution low-dose photon-counting CT of the breast. Eur Radiol 2017;27:1081–6. 27306559 10.1007/s00330-016-4459-3

[31] Ekpo EU Ujong UP Mello-Thoms C. Assessment of interradiologist agreement regarding mammographic breast density classification using the fifth edition of the BI-RADS atlas. AJR Am J Roentgenol 2016;206:1119–23. 26999655 10.2214/AJR.15.15049

[32] Winkel RR von Euler-Chelpin M Nielsen M. Inter-observer agreement according to three methods of evaluating mammographic density and parenchymal pattern in a case control study: impact on relative risk of breast cancer. BMC Cancer 2015;15:274. 25884160 10.1186/s12885-015-1256-3 PMC4397728

[33] Ciritsis A Rossi C Vittoria De Martini I. Determination of mammographic breast density using a deep convolutional neural network. Br J Radiol 2019;92:20180691. 30209957 10.1259/bjr.20180691 PMC6435091

